# A comparative study on trocar configurations and the use of steerable instruments in totally extraperitoneal inguinal hernia surgery training

**DOI:** 10.1007/s00464-025-11541-7

**Published:** 2025-02-03

**Authors:** M. E. C. M. van de Pas, R. R. Postema, H. P. Theeuwes, J. W. A. Klok, M. Rahimi, C. Verhoef, Tim Horeman

**Affiliations:** 1 Department of Biomechanical Engineering, Faculty of BioMechanical Engineering, Delft University of Technology, Mekelweg 2 (Building 34), 2628CD Delft, The Netherlands; 2 Department of Surgery, Erasmus MC, Rotterdam, The Netherlands; 3 Department of Surgery, Amsterdam UMC – VU University Medical Center, Amsterdam, The Netherlands; 4 Amsterdam Skills Center, Amsterdam, The Netherlands; 5 Erasmus MC SkillsLab, Rotterdam, The Netherlands; 6 Department of Surgery, ETZ, Tilburg, The Netherlands

**Keywords:** Hernia repair, TEP, Steerable instruments, Laparoscopy, Midline configuration, SATA

## Abstract

**Background:**

Totally extraperitoneal (TEP) inguinal hernia surgery is a commonly performed but technically challenging procedure with a long learning curve. As TEP can be executed using two different trocar placements: a midline or a triangular configuration, the question remains which one is technically easier to master.

**Methods:**

In a multicenter crossover-study, medical students were randomised into two groups and executed tasks on a box trainer that measured time, volume and force parameters. Additionally, the study assessed whether the SATA instrument, a steerable laparoscopic instrument that articulates the instrument’s tip, would reduce the difficulty of performing the tasks in the midline configuration. After training, all participants executed a first experiment using both trocar configurations, followed by a second experiment executed with steerable and non-steerable instruments in the midline configuration. Subjective and objective performances per condition and learning curves were assessed.

**Results:**

Participants were faster and showed lower peak forces in the triangulated configuration. Learning curve analysis showed a positive improvement in time and path length in the midline configuration. Although participants rated ergonomics and intuitiveness similarly between the instruments, they found the task easier with the SATA instruments, ranking the added value of the steering function as 5 out of 5. Objectively, time and path length showed no significant differences while exerted forces were lower when using conventional instruments.

**Conclusion:**

Although the midline configuration is preferred in terms of comfort and posture, the findings indicate that, for inexperienced practitioners, performing TEP surgery in midline configuration is both subjectively and objectively more challenging, highlighting the need for extensive training to overcome its difficulties and possibly shorten its learning curve. Although instruments with additional steering functions were preferred over conventional instruments in the more challenging midline configuration, additional steering complexity did not result in better parameter outcomes, showing the need for more extensive training.

**Supplementary Information:**

The online version contains supplementary material available at 10.1007/s00464-025-11541-7.

Worldwide, over 20 million inguinal hernia surgeries are performed each year, ranking it as one of the most common procedures globally [1]. A laparoscopic approach is often used in high-resource countries, such as in the Netherlands where 53% of all inguinal hernia repairs are performed laparoscopically [2]. The most established laparoscopic approaches are the transabdominal preperitoneal (TAPP) and totally extraperitoneal (TEP) approaches, of which the European Hernia Society guideline favours a TEP [3]. TEP surgery, however, is technically challenging and is known to have a very long learning curve whereby, even after 400 procedures, there is still an increase in the quality of surgery performed [4]. This learning period is associated with a higher rate of serious complications [3], indicating the need for improvement of knowledge, training and perhaps technical approaches. This research aims to address two of these needs through the following investigations.

## Trocar placement in TEP surgery

Firstly, this study will compare two different trocar placements in TEP surgery. Conventional TEP surgery requires a camera (positioned sub-umbilically) and two additional trocars, which can be positioned either in the midline or in a triangulated configuration with one in the midline and one laterally [5]. Although both methods are practised in TEP surgery, a comparative analysis between the two is lacking. Commonly, triangulation is considered a basic principle of laparoscopic port placement, being more intuitive with an instrument on the left and the right side of the point of view [6, 7]. Also the triangular positioning gives a better possibility of traction and countertraction movements. However, in TEP surgery, the triangulated positioning of the trocars requires the surgeon to stretch and reach across the body of the patient to the lateral trocar, which is a less ergonomic working position. In the midline configuration, standing upright and without rotation in the surgeon’s posture parallel to the body of the patient diminishes the physical workload. Additionally, the midline configuration is advantageous in two-sided TEP surgeries, as it could eliminate the need for an extra incision when approaching the contralateral side. This research aims to investigate the respective difficulties and conveniences of both positions for inexperienced practitioners.

## Shaft actuated tip articulated (SATA) instruments

**Fig. 1:**
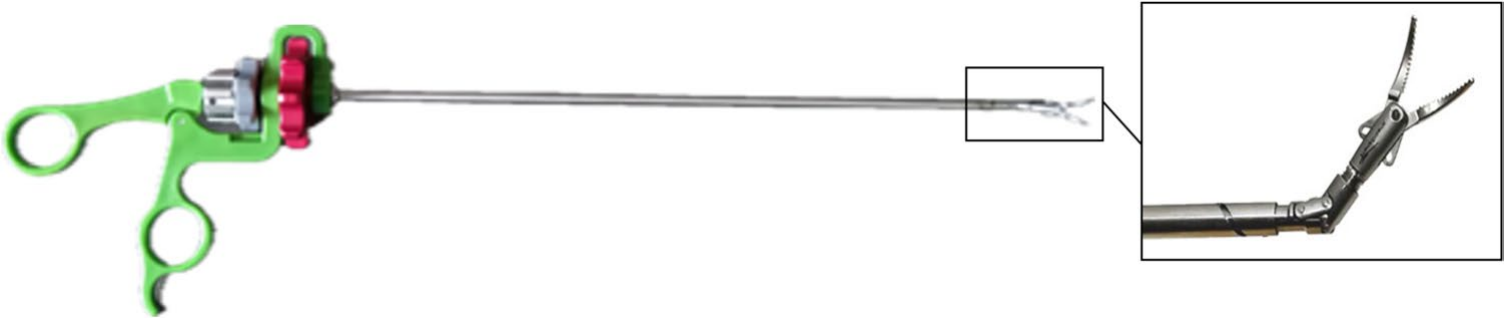
Left: a picture of the full SATA instrument, featuring the newly designed handle. Right: a picture of the tip of the SATA instrument with a rotatable end-effector while articulated [8]

Secondly, the difficulties of TEP surgery highlight the potential benefits of technical innovation. Therefore, this procedure will be used as a reference to pre-clinically test a new device: the Shaft Actuated Tip Articulated (SATA) instruments [8] (Fig. 1). The SATA steering technology was created as a new basis for sustainable (robot) instruments that should foster more laparoscopic instrument functionality on a global level. These reusable, steerable laparoscopic instruments provide two additional degrees of freedom, enabling up to 80° articulation of the instruments tip. This offers extra dexterity compared to conventionally used non-steerable laparoscopic instruments. Also, the instruments differ from other steerable options since they operate without the use of cables for tip actuation. Instead, they use rigid tubes, thereby providing comparable haptic feedback to conventional instruments. Using these rigid tubes also provides a smaller bending radius and a hollower shaft that allows for the integration of additional functionalities. Previous pre-clinical tests have evaluated the learning curves and shown positive reactions of surgeons to the use of the SATA steering technology when implemented in a handheld device [9]. For this study, the handles of the SATA instruments resembled the appearance and tactile experience of standard laparoscopic instruments but with the extra functionality of steering of the tip [9]. The question is whether these instruments can benefit the learning- and execution of TEP surgery, maybe especially so in a less than optimal position of the instruments like in the midline position described above. With the added steering and “wrist-like movement” capability of the SATA instruments the traction and countertraction actions that with conventional instruments are more easily possible in the triangular position, could also be more attainable in the midline position of the trocars. Therefore, further objective measurements are necessary to assess their performance. Hence, this research will also compare the use of conventional laparoscopic instruments to the newly designed SATA instruments in the challenging context of TEP surgery with the added difficulty of the midline positioning of the trocars.

## Materials and methods

### Study design and participants

This multi-centre randomised crossover study was conducted between February 2024 and July 2024.

The study enrolled medical students from the Erasmus Medical Centre (Rotterdam, the Netherlands) and the Amsterdam University Medical Centre (Amsterdam, the Netherlands). Participants were recruited voluntarily through announcements that were distributed via medical student associations and programs in Amsterdam and Rotterdam. A pre-test survey was conducted to obtain informed consent and baseline characteristics (Supplemental File A). All participants completed a laparoscopic training module before executing the tests.

### Hardware and systems

**Fig. 2:**
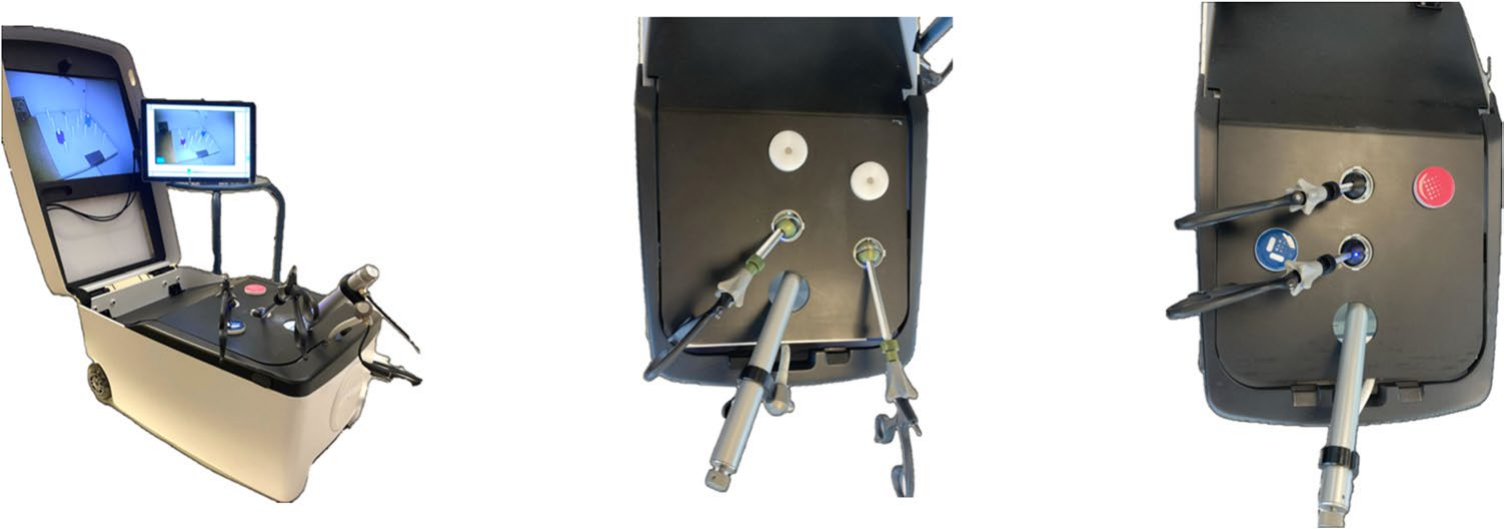
Left: a picture of the setup featuring the box trainer, newly designed panel, the newly located screen and two Maryland graspers. Middle: a picture of the designed panel with the trocars and camera in triangular configuration. Right: a picture of the designed panel with the trocars and camera in the midline

For the experiments, the Lapron portable laparoscopic boxtrainer [10] was used, equipped with the ForceSense measuring system (both: ForceSense B.V., Delft, The Netherlands) [11]. This system measures all interaction forces in 3D and tracks the motion of the instrument tips during the execution of a task. The training and tests were conducted using standard curved Maryland grasping forceps and the SATA curved grasping forceps (Fig. 1). Two new top panels were designed and fabricated out of Polymethyl methacrylate (PMMA) for the box trainer. One allowed for trocar placement in the midline, and the other in a triangulated configuration (Supplemental File B), mimicking the situation during TEP surgery [5]. Additionally, one of the screens of the box trainer was moved to the side to allow participants to look straight ahead while positioned on the side of the box trainer, simulating inguinal hernia surgery [12] (Fig. 2). Lastly, the task-board (placed in the box trainer, normally on the centre of the back wall) was elongated laterally. This allowed for placement of the task in a position corresponding to the “right groin”, the side that inguinal hernias are most prevalent [13]. Although the tests were conducted in two different trocar configurations, equal force and motion measurement capabilities were ensured.

### Study protocol

**Fig. 3:**
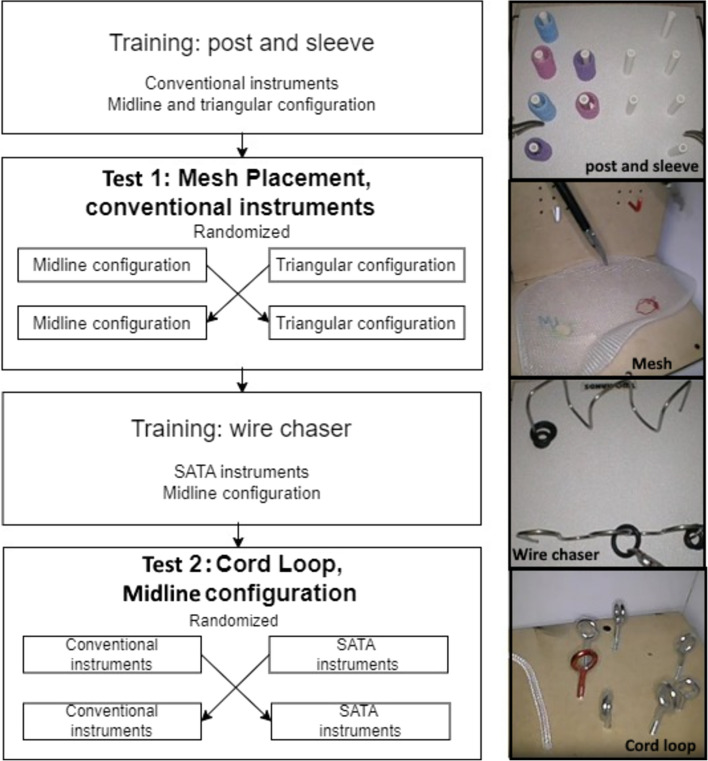
Flowchart of the study protocol

The crossover design is shown in Fig. 3. The participants were randomised into two groups using https://www.randomizer.org/. Firstly, all participants completed the first training, performing it with the trocars in the midline and triangulated configuration. Subsequently, the first Test 1 (Mesh Placement task) was conducted in both configurations.

Secondly, all participants performed the second training, using a set of two conventional graspers and a set of one conventional grasper and a SATA grasper in their dominant hand. Then, the second Test 2 (Cord Loop task) was performed using both sets of instruments. All tasks were performed twice under both conditions.

Group 1 completed the Test 1 in triangular configuration followed by midline configuration and Test 2 with conventional instruments followed by the SATA instruments. For Group 2, the conditions of Test 1 and Test 2 were reversed.

### Tasks

For the first and second training, two validated training tasks were used: the Post and Sleeve task and the Wire chaser task, respectively [14].

Two new Tests were designed to address the research questions. The first task for Test 1 was designed to compare trocar placement in the midline to placement in a triangulated configuration. This task, the Mesh Placement task, requires a participant to place an inguinal hernia mesh (Dextile™ Anatomical Mesh, right side, 15 × 10 cm, Medtronic Eindhoven, The Netherlands) with two added holes onto two hooks with corresponding colours and afterwards place it back in the starting position. This task was performed with conventional instruments.

The second new task was developed for Test 2 to compare conventional non-steerable instruments to the SATA instruments. This task, named the Cord Loop task, involves threading a cord counterclockwise through holes that alternate in height and orientation. This task was based on the previously validated pattern cut task [15]. The task was performed in midline configuration, as this configuration was hypothesised to be more challenging while providing a more comfortable posture for the practitioner. As the SATA steering functionality was already previously evaluated by our team in the triangular configuration within a Lapron boxtrainer with ForceSense technology on a 3D FLS task with good results [9], we chose to further investigate the added value of instrument steering in the more challenging midline setting only.

To ensure a proper understanding of the tasks, participants first watched an instructional video featuring an experienced practitioner completing the task. Visualisation of the tasks can be found in Supplemental File C.

### Software and data collection

The ForceSence system recorded real-time video, force, time and volume parameters for each task attempt. These were stored in an online server (ForceSense B.V., Medishield, Delft, The Netherlands) [11]. Participants also filled out a questionnaire after each Test to score and describe their experiences. The questions were answered using a 1–5 Likert scale, and there were also open questions for feedback (Supplemental File D). The resulting data was analysed using IBM SPSS (version 29.0, SPSS, Inc., Chicago IL, USA). Solid Edge (version 2021, Siemens Digital Industries Software, Plano, TX, USA) was used for the design of the box trainer panels and tasks.

### Study parameters

The main objective parameters for both studies included the time taken to complete the task (in seconds), the maximum force applied to the task board (in Newton), the mean force exerted by the instruments on the task board during periods when force is not zero (in Newton) and the cumulative path lengths of the left and right instruments (in mm). These parameters have been shown to be the most indicative and discriminating for performance on tasks in a laparoscopic box trainer [11, 16]. For practical reasons, the maximum time allowed for Test 1 and 2 completion was set at 400 s each. Trials exceeding this limit were marked as ‘unfinished’.

The primary subjective parameters for comparing trocar placement were the difficulty of completing the Tests, the amount of overview and the comfort of the posture of the participant in each configuration.

For the comparison between conventional and SATA instruments the main subjective parameters were the difficulty of completing Test 2, the ergonomics of the instrument and the perceived added value of steerability during the experiment.

Secondarily, the objective parameter differences between the first and second attempts per task and condition were compared to assess learning curves and effects. Also, the effect of the order in which participants used the two different trocar configurations and instruments was evaluated. To do so, a comparison was made for each condition between the participants who executed the task under this condition first or after completing the task using the other configuration or instruments.

Lastly, the comprehensibility and feasibility of the Tests 1 and 2 were assessed by analysing and comparing the answers to two questions from the questionnaire. These questions asked participants if the tasks were easy to understand and achievable within the given time. An average score above 3 on the 1–5 Likert scale was considered sufficient.

### Statistical analysis

Normality tests were performed for all parameters. Regardless of sample size, parametric tests (either paired or unpaired *t* tests) were validated using non-parametric tests (Mann–Whitney *U* or Wilcoxon signed rank if paired) when normality was questionable to ensure for powerful yet robust statistical analysis. A *p* value < 0.05 was considered significant. If many of the trials exceeded the time limit, a Tobit regression was performed on the time parameter to correct for censoring. This was combined with a Wilcoxon signed-rank test to account for potential non-normality and the presence of outliers in the data.

## Results

32 Participants were enrolled (15 in Amsterdam and 17 in Rotterdam). They were randomised into two groups of 16 that had comparable baseline characteristics (age, sex, dominant hand, study year, laparoscopic experience and weekly time spent on playing video games or a musical instrument) (Supplemental File E).

In total, the participants performed 256 training trials and 256 Test trials that were recorded and analysed. There were 37 test trials marked as unfinished which were all Cord Loop tasks.

### Questionnaire responses trocar placement

Table 1 shows the results of the questionnaires comparing triangular to midline configuration (Supplemental File D). Significant differences were found for the perceived difficulty, overview and comfort of the posture. Detailed results and further analysis are shown in Supplemental File F. In the open questions, 26 participants mentioned a more comfortable posture in the midline configuration. Also, some commented that, in triangular configuration, it was easier to distinguish the left from the right instrument (20 mentions), instruments crossed each other less often (7 mentions) and instruments obstructed the camera view less often (6 mentions) (Supplemental File G).

**Table 1:** Results and analysis of questionnaire responses on trocar placement

Question	Triangular*	Midline*	Sign.**
Easiness of the task	4 (2)	3 (2)	** < 0.001** (< 0.001)
Overview of task and instruments	4 (1)	3 (3)	** < 0.001** (< 0.001)
Comfortable posture	3 (3)	4 (2)	** < 0.001** (< 0.001)
Understandable task	5 (1)	5 (1)	0.325 (0.317)
Task is achievable within the time	5 (1)	5 (3)	0.070 (0.068)

Bold values reflect the significance between groups (*p* < 0.05)

*Median (range), **unpaired *t* test (Wilcoxon signed-rank)

### Questionnaire responses SATA instruments

The results for questions about the Cord Loop task in Test 2, comparing conventional and SATA instruments, are shown in Table 2. A significant difference was found for the perceived difficulty of the task. Detailed results and further analysis are shown in Supplemental File F. When asked if the steering function of the SATA instruments was of added value, the response median was 5 out of 5 (range 2). In the open questions (Supplemental File G), 25 participants mentioned being able to reach more angles with the SATA instrument. However, 14 participants mentioned that manipulating the tip of the SATA instruments required more thinking than the conventional instruments. Some participants provided feedback on the design of the SATA instruments, such as the need for more degrees of rotation of the tip (currently fixed at 360°) (4 mentions).

**Table 2:** Results and analysis of questionnaire responses comparing instrument-type

Question	Conventional *	SATA*	Sign.**
Easiness of the task	3 (4)	4 (4)	**0.011** (0.020)
Easiness of use of instrument	4 (3)	4 (4)	0.845 (0.868)
Intuitiveness of use of instrument	4 (3)	4 (3)	0.521 (0.518)
Ergonomics of instrument	4 (3)	4 (4)	0.737 (0.657)
Understandable task	5 (1)	5 (1)	0.057 (0.059)
Task is achievable within the time	4 (4)	4 (4)	0.234 (0.281)

Bold value reflects the significance between groups (*p* < 0.05)

*Median (range), **unpaired *t* test (Wilcoxon signed-rank)

### Objective measurements trocar placement

**Fig. 4:**
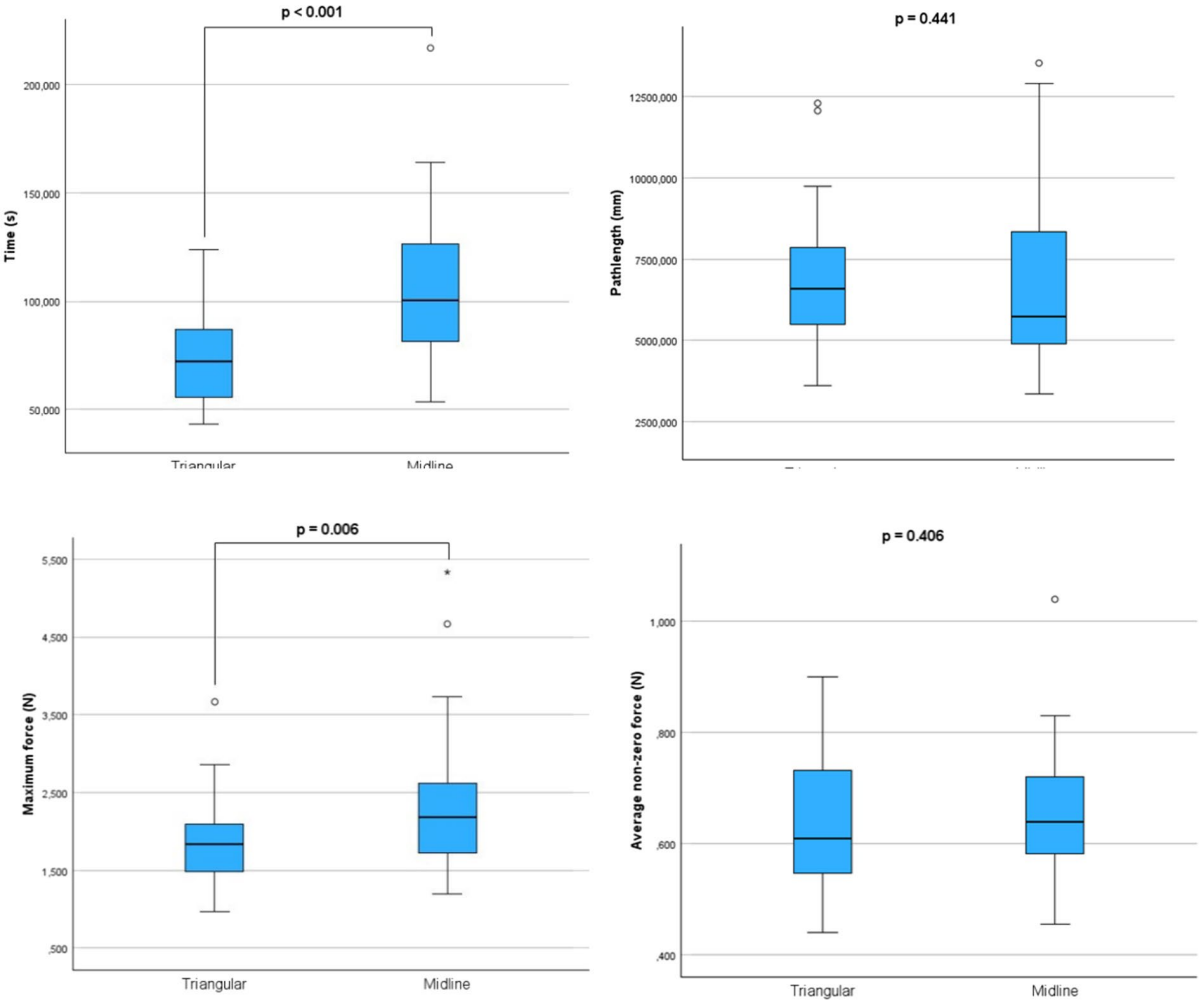
Boxplots of the objective parameters (time, path length, maximum force and average non-zero force) resulting from the Mesh Placement task in triangular and midline configuration. Significance shown by *p* value resulting from 2-tailed *t* tests

Figure 4 shows the boxplots of the time to completion, path length, maximum force and average non-zero force of the Mesh Placement task of Test 1 in triangular and midline configurations. For both conditions, time and average non-zero force measurements were normally distributed. For path length and maximum force, a Wilcoxon signed-rank was performed to validate the paired *t* test. The time and maximum force were significantly lower in triangle configuration. No significant differences were found in path length and the average non-zero force. Detailed results and normality tests and additional analysis are stated in Supplemental File H.

### Objective measurements SATA instruments

**Fig. 5:**
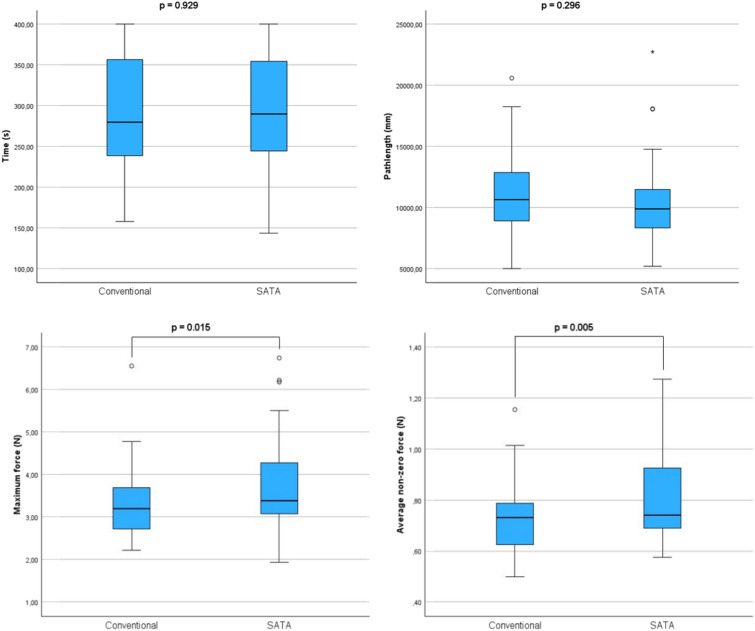
Boxplots from the objective parameters (time, path length, maximum force and average non-zero force) resulting from the Cord Loop task with conventional and SATA instruments. Significance shown by *p* value resulting from 2-tailed paired *t* tests and Tobit regression for the time parameter

Figure 5 shows the boxplots of the time to completion, path length, maximum force and average non-zero force of the Cord loop task of Test 2 executed with conventional and SATA instruments. For the maximum force parameter, both conditions were normally distributed. On the other parameters, parametric tests were validated by a Wilcoxon signed-rank. There were no significant differences found in time and path length. The maximum force and average non-zero force were found to be lower when using conventional instruments. For the time parameter, a Tobit regression was performed due to the large number of trials exceeding the time limit. Detailed results, illustrations, normality tests and additional analysis are stated in Supplemental File H.

### Learning curves and effects

Table 3 shows the comparison of the first and second attempts at the Mesh Placement task of Test 1 for each trocar configuration. A significant improvement was found in the time and path length in the midline configuration. Table 4 subsequently shows the same analysis for the two performances of the Cord Loop task of Test 2 per instrument. The average non-zero force was significantly improved when using the SATA instrument. For both tasks, boxplots of these analysis are shown in Supplemental File I.

**Table 3:** Comparison of first and second attempt at the Mesh Placement task per trocar configuration

	First attempt mean	Second attempt mean	Sign.*
Time (s), triangular	78.90	70.03	0.090
Time (s), midline	118.58	96.13	**0.039**
path length (mm), triangular	6933.34	6967.69	0.479
path length (mm), midline	7221.79	5974.99	**0.046**
Maximum force (*N*), triangular	1.88	1.84	0.408
Maximum force (*N*), midline	2.42	2.31	0.711**
Average non-zero force (*N*), triangular	0.62	0.66	0.104
Average non-zero force (*N*), midline	0.64	0.67	0.103

Bold values reflect the significance between groups (*p* < 0.05)

*Paired *t* test, **Wilcoxon signed-rank

**Table 4:** Comparison of first and second attempt at the Cord Loop task per instrument

	First attempt mean	Second attempt mean	Sign.*
Time (s), conventional	307.91	270.50	0.139**
Time (s), SATA	298.66	289.56	0.527**
Path length (mm), conventional	11,860.23	10,812.13	0.106
Path length (mm), SATA	10,456.01	10,763.17	0.331
Maximum force (*N*), conventional	3.41	3.28	0.284
Maximum force (*N*), SATA	3.55	3.98	0.093
Average non-zero force (*N*), conventional	0.73	0.74	0.337
Average non-zero force (*N*), SATA	0.84	0.77	**0.041**

Bold value reflects the significance between groups (*p* < 0.05)

*Paired *t* test, **Tobid regression

The results of the evaluation of the effect of the order in which participants completed both conditions for each Test are shown in Supplemental File J. This showed no significant differences between participants performing a Test under a condition before and after performing that Test under the other condition.

### Task comprehensibility and feasibility

As shown in Tables 2 and 3, the median rating of understandability of the task was 5 out of 5 for all conditions in both Tests. Participants rated the extent to which the Mesh placement task in Test 1 was achievable within the time a 5 out of 5 for both trocar placements. For the Cord Loop task of Test 2 this was a 4 out of 5 for both instruments. In neither of the tasks, significant differences between the answers to these questions were observed for the different trocar configurations or instruments. An illustration of the responses and their distribution is shown in Supplemental File K.

## Discussion

The comparable baseline characteristics between the two study groups ensured a robust and fair statistical analysis. The high scores for task comprehensibility and feasibility under all conditions suggest that the tasks of both Tests were well-understood and realistically achievable. These results support the design of the test setup, tasks and instructions. This contributes to a fair comparison between the conditions and the overall validity of the study.

### Triangulated vs. midline trocar positioning

Subjectively, participants rated the midline configuration as being more difficult, complex and providing less overview of the task and the instruments than the triangular configuration. This assessment was supported by the objective results showing that the task was performed slower and with higher peak forces in the midline configuration than the triangular configuration. We see that overall, participants show less task time in executing the task in the triangular approach. Indicating that they experience less difficulties in translating the information on the screen to the in vitro motor controls of the instruments. These findings concur with the common assumption that trocar placement in triangulation aligns with human anatomy, making it more intuitive [6, 7]. It suggests that the learning curves before mastering the skills in the triangular approach will be shorter compared to the midline approach. However, the results from the questionnaire showed a clear preference for the midline configuration in terms of comfort and posture, likely because it allows for a more upright position (and not a twisted or rotated one) with two instruments positioned closer to the operator. Given the importance of good posture during surgery [7], this advantage is a significant benefit, even if the midline configuration proves more challenging for (learning the) task execution. The observed learning curves for the midline configuration suggest a promising potential for improvement with additional training. No distinct difference was found in performances when comparing the two configurations’ learning order. This indicates that proficiency in one configuration does not necessarily translate to the other, stressing the need for training in both configurations separately. These results provide useful insights for the education and guidance of less-experienced practitioners. They highlight the added value and challenges of the midline configuration, underscoring the need for more extensive training to maximise its ergonomic benefits and overcome its initial difficulties.

### Conventional vs. SATA instruments

In the questionnaires, participants showed a preference for the SATA instruments over conventional instruments, finding the tasks easier to perform and valuing the steering function as an additional feature, while other aspects were rated similarly. However, this preference did not translate to objective parameters, with the SATA instruments performing equally in terms of time and path length but demonstrating higher force parameters compared to conventional instruments. This discrepancy could be caused by a difference in learning curves. While the learning curve of SATA instruments has been researched before [6], it has not been compared to that of conventional instruments. The additional features and options provided by the SATA instruments may contribute to a steeper initial learning curve. A support for this theorem is that participants noted that using the SATA instruments required more cognitive effort due to the additional steering function, suggesting that with more practise, users might become more proficient. This potential for improvement is highlighted by significant improvements observed in force parameters between the first and second attempts with the SATA instruments. Thus, these findings indicate that with additional training, the objective performance of SATA instruments could improve, aligning with the participants’ subjective preference for these instruments. These insights are a valuable addition to existing research. This study is the first to assess the performance of SATA instruments on parameters such as tissue handling forces and path length of the instrument tips, providing a clearer understanding of their benefits and challenges. Secondly, the study validates the ergonomics of the new design of the SATA instruments, which was perceived to be equal to the current gold standard.

### Observations from this study on training and performing TEP surgery

Training TEP surgery has always been a hands-on experience without widely available ex-vivo training possibilities. Our newly developed training set-up could aid in training this challenging type of surgery while simultaneously measuring the progression of the training to a level whereby trainer and trainee are both comfortable to perform side-by-side in vivo surgery. Also, more advanced (but more comfortable) set ups, like the midline trocar configuration with their initial drawbacks can be experienced and trained before switching to clinical surgery. Our SATA instruments take some training to get used to but show promise in more quickly overcoming some of the drawbacks of the midline trocar placement and enhancing the dexterity needed during TEP surgery.

### Limitations and future work

This study provided valuable initial insights regarding the two trocar configurations. However, the extent to which the test setup and the short task of Test 1 resemble the full TEP procedure is limited. Therefore, future studies should evaluate practitioners’ subjective experiences during in vivo surgery and assess objective parameters such as operation time and complications but could also study objective assessment of posture ergonomics of participants in the different trocar configurations [17]. Also, further insights for training could be gathered by identifying the specific difficulties of TEP surgery, particularly in the midline configuration, through the evaluation of video footage from box trainers or real procedures. This could provide personalised feedback on an individual level, possibly facilitating quicker improvement and shortening the established long learning curve [3].

Due to the multitude of tasks required for participants’ training and testing of both research questions, the time available to test each condition was limited. This was done to avoid fatigue and ensure that participants could maintain proper focus. However, the limited number of repetitions per instrument did not allow for an analysis of the full learning curve or a comparison of the instruments after reaching the learning plateau. Consequently, future research should focus on extending the testing period to thoroughly evaluate the learning curves and performance of the instruments over a more extended timeframe.

Another limitation was the time restriction per Test due to the limited time per participant. This did not affect the Mesh Placement task, as all participants completed it within the available time. However, the Cord Loop task, designed to test precise three-dimensional work in the midline configuration, proved more difficult. Many participants could not finish one or more attempts, posing a problem for the time parameter. To address this, Tobit regression and Wilcoxon signed-rank tests were used to ensure robust statistics. This likely did not impact the overall results significantly, given the minimal absolute differences in the time results. For other parameters, censoring had less impact as they were less dependent on the time spent on the task.

Finally, the questionnaires provided valuable feedback on the design of the SATA instruments, which offers insights for potential enhancements. One example was the feedback that some participants felt limited by the maximum of 360° rotation of the tip. This stood out when compared to the conventional instruments that are able to rotate infinitely.

## Conclusion

The findings indicate that, for inexperienced practitioners, performing TEP surgery in midline configuration is both subjectively and objectively more challenging compared to triangulated trocar placement. However, the midline configuration is preferred in terms of comfort and posture and advantageous in two-sided TEP surgeries, as it eliminates the need for an extra incision when approaching the contralateral side. This highlights the need for more extensive training to overcome its difficulties and possibly shorten its learning curve.

Regarding instrument use, medical students favour SATA instruments over conventional instruments in the challenging setting of TEP surgery in midline configuration. They perceive the steering function to be of added value. However, the objective results do not support this preference. Acknowledging the identified, promising learning effects, further research is needed to compare the instruments after more extensive training to account for potential differences in learning curves.

## Supplementary Information

Below is the link to the electronic supplementary material.

Supplementary file1 (DOCX 42 KB)

Supplementary file2 (DOCX 160 KB)

Supplementary file3 (DOCX 17 KB)

Supplementary file4 (DOCX 42 KB)

Supplementary file5 (DOCX 14 KB)

Supplementary file6 (DOCX 64 KB)

Supplementary file7 (DOCX 726 KB)

Supplementary file8 (DOCX 22 KB)

Supplementary file9 (DOCX 15 KB)

Supplementary file10 (DOCX 52 KB)

Supplementary file11 (DOCX 17 KB)
