# Supplementary material for: A comparative study on trocar configurations and the use of steerable instruments in totally extraperitoneal inguinal hernia surgery training
Source: Surg Endosc. 2025 Feb 3;39(3):2080–90. doi: 10.1007/s00464-025-11541-7 (PMC11870937; doi:10.1007/s00464-025-11541-7)
Supplement: Supplementary file 1 — Supplementary file1 (DOCX 42 KB) [file 464_2025_11541_MOESM1_ESM.docx]

# Supplemental file H: objective parameter results and analysis

## Trocar placement: triangular and midline

Table 12 shows the descriptives and the results of normality tests for the objctive parameters (time, path length, maximum force and avegage non-zero force) measured in the Mesh Placement task to compare triangular and midline trocar configuration. Futheremore, table 13 shows the results of statistical comparison betweem trian- gular and midline configuration.

**Table 12**: objective results Mesh Placement task

|  | Mean | Median | Range | IQR | Skewnes | Kurtosis | Norm* |
| --- | --- | --- | --- | --- | --- | --- | --- |
| Time, triangular (s) | 74.47 | 72.00 | 81 | 34.50 | 0.713 | -0,159 | 0.063 |
| Time, midline (s) | 107.35 | 100.5 | 163.50 | 52.00 | 0.904 | 1.259 | 0.144 |
| path length, triangular (mm) | 6950.5 | 6595.2 | 8696.6 | 2437.2 | 1.049 | 0.991 | 0.022 |
| path length, midline (mm) | 6542.4 | 5746.9 | 10186.4 | 4424.5 | 1.133 | 0.883 | 0.004 |
| Maximum force, triangular (N) | 1.87 | 1.835 | 2.695 | 0.625 | 1.122 | 2.184 | 0.061 |
| Maximum force, midline (N) | 2.36 | 2.18 | 4.135 | 0.925 | 1.571 | 2.877 | 0.001 |
| Average non-zero force, triangu-  lar (N) | 0.64 | 0.61 | 0.46 | 0.205 | 0.507 | -0.778 | 0.106 |
| Average non-zero force, midline  (N) | 0.656 | 0.64 | 0.585 | 0.140 | 1.118 | 2.441 | 0.057 |

*Normality via Shapiro-Wilk

**Table 13**: results statistical analysis Mesh Placement task

|  | Triangular mean | Midline mean | sign*. |
| --- | --- | --- | --- |
| Time to completion (s) | 74.47 | 107.35 | **<0.001** |
| path length (mm) | 6950.5 | 6542.4 | 0.441 (0.264) |
| Maximum force (N) | 1.87 | 2.36 | 0.006 (0.004) |
| Average non-zero force (N) | 0.64 | 0.656 | 0.406 |

*unpaired t-test (Wilcoxon signed-rank if performed)

## Instrument comparison: conventional and SATA

Table 14 shows the descriptives ans results of normality tests for the objctive parameters (time, path length, maximum force and avegage non-zero force) measured in the Cord Loop task comparing conventional and SATA instruments. Table 15 shows the results of statistical comparison between these instruments. Also, the frequency table of the time parameter is shown (with exceeding times censored at the time limit of 400s) (*Figure 14* )

**Table 14**: objective results Cord task

|  | Mean | Median | Range | IQR | Skewnes | Kurtosis | Norm* |
| --- | --- | --- | --- | --- | --- | --- | --- |
| Time, conventional (s) | 289.2 | 289.2 | 242.0 | 122.0 | 0.056 | -1.013 | 0.064 |
| Time, SATA (s) | 294.1 | 290.0 | 256.0 | 115.4 | -0.276 | -0.901 | 0.251 |
| path length, conventional (mm) | 11336.2 | 10670.2 | 15553.7 | 4193.5 | 0.705 | -0.002 | 0.162 |
| path length, SATA (mm) | 10609.6 | 9896.5 | 17532.7 | 3321.61 | 1.495 | 2.920 | 0.002 |
| Maximum force, conventional  (N) | 3.34 | 3.20 | 4.34 | 1.04 | 1.54 | 3.80 | 0.003 |
| Maximum force, SATA (N) | 3.77 | 3.38 | 4.81 | 1.29 | 0.885 | 0.148 | 0.017 |
| Average non-zero force, conven-  tional (N) | 0.738 | 0.732 | 0.66 | 0.17 | 0.942 | 1.061 | 0.070 |
| Average non-zero force, SATA  (N) | 0.799 | 0.740 | 0.70 | 0.24 | 1.143 | 0.974 | 0.004 |

*Normality via Shapiro-Wilk

**Table 15**: results statistical analysis Cord Loop task

|  | Conventional mean | SATA mean | sign*. |
| --- | --- | --- | --- |
| Time to completion (s) | 289.2 | 294.1 | 0.929 (0.631) |
| path length (mm) | 11336.2 | 10609.6 | 0.296 (0.254) |
| Maximum force (N) | 3.34 | 3.77 | 0.015 |
| Average non-zero force (N) | 0.738 | 0.799 | 0.005 (0.004) |

*unpaired t-test (Wilcoxon signed-rank if performed)


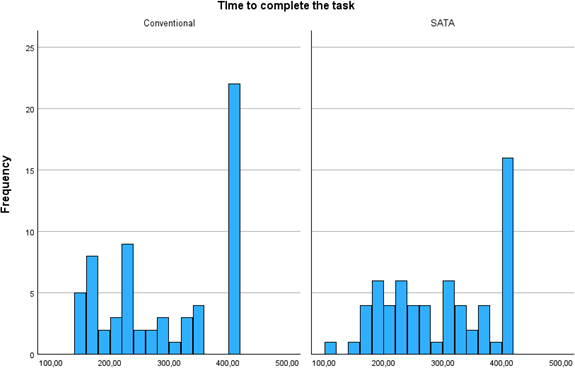


**Figure 14:** Frequency of time to complete the Cord Loop task with conventional and SATA instruments
