# Supplementary material for: A comparative study on trocar configurations and the use of steerable instruments in totally extraperitoneal inguinal hernia surgery training
Source: Surg Endosc. 2025 Feb 3;39(3):2080–90. doi: 10.1007/s00464-025-11541-7 (PMC11870937; doi:10.1007/s00464-025-11541-7)
Supplement: Supplementary file 2 — Supplementary file2 (DOCX 160 KB) [file 464_2025_11541_MOESM2_ESM.docx]

# Supplemental file I: figures of learning curve analysis

## Trocar placement: triangular and midline (Mesh Placement task)


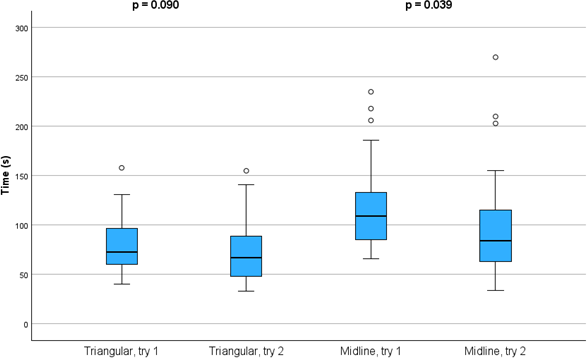


Figure 15: Comparison of time parameter between first and second try for each trocar configuration


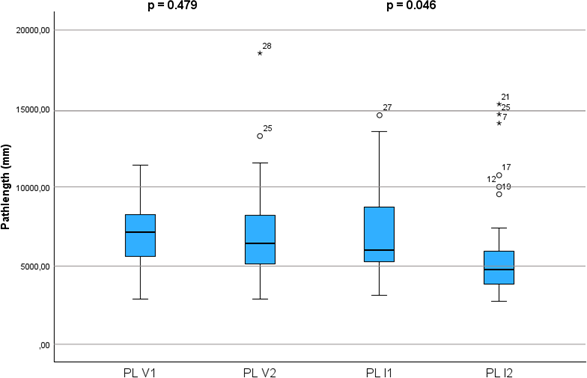


Figure 16: Comparison of path length parameter between first and second try for each trocar configuration


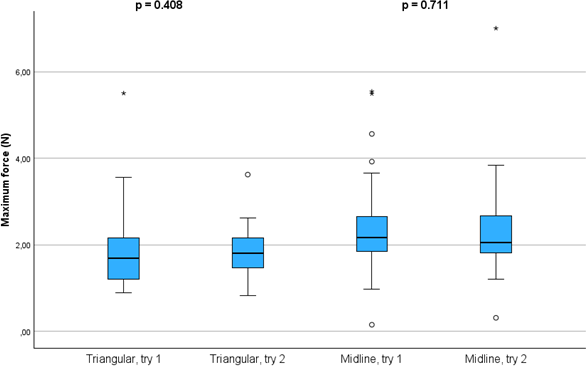


Figure 17: Comparison of maximum force between first and second try for each trocar configuration


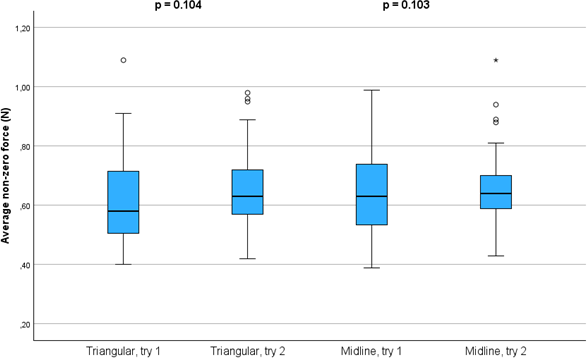


Figure 18: Comparison of average non-zero force between first and second try for each trocar configuration

## Instrument: conventional and SATA (Cord Loop task)


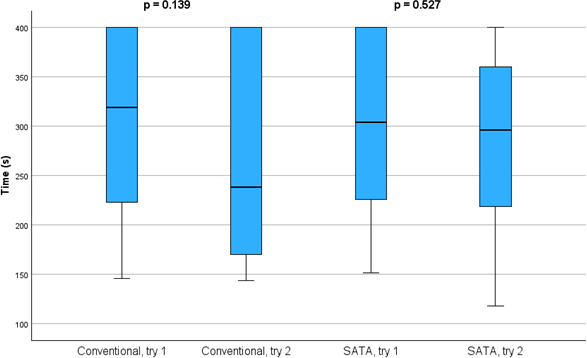


Figure 19: Comparison of time parameter between first and second try for each instrument


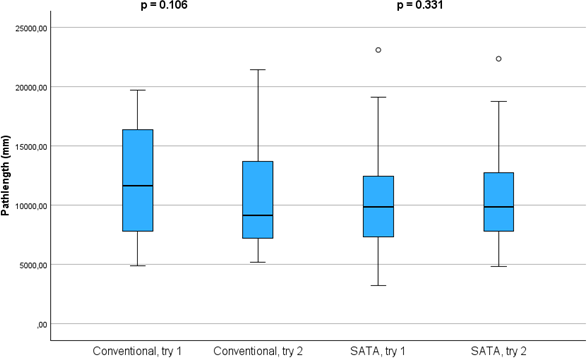


Figure 20: Comparison of path length parameter between first and second try for each instrument


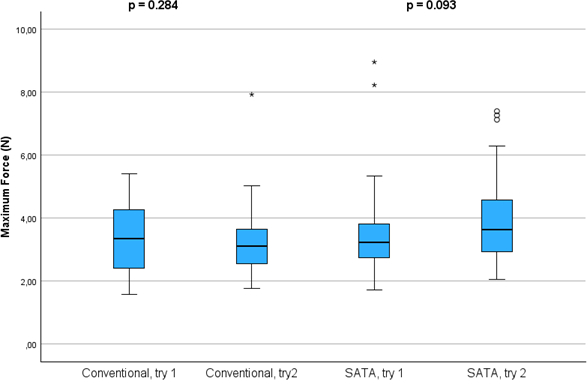


Figure 21: Comparison of maximum force between first and second try for each instrument


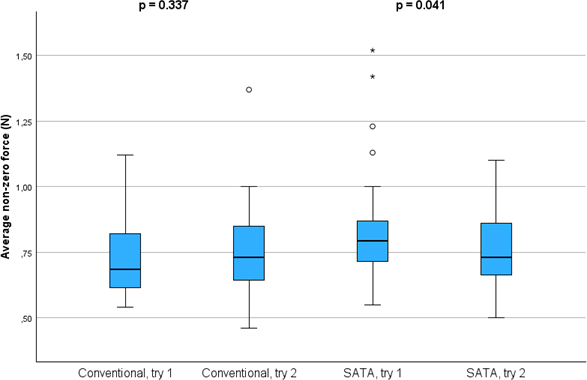


Figure 22: Comparison of average non-zero force between first and second try for each instrument
