# Supplementary material for: A comparative study on trocar configurations and the use of steerable instruments in totally extraperitoneal inguinal hernia surgery training
Source: Surg Endosc. 2025 Feb 3;39(3):2080–90. doi: 10.1007/s00464-025-11541-7 (PMC11870937; doi:10.1007/s00464-025-11541-7)
Supplement: Supplementary file 3 — Supplementary file3 (DOCX 17 KB) [file 464_2025_11541_MOESM3_ESM.docx]

# Supplemental file J: effect of order of condition randomization

Table 16 shows the results of statistical analysis of the effect of performing the Mesh Placement task in a trocar configuration before or after performing this task in the other trocar configuration. This is shown for each of the main objective parameters. Table 17 shows the same analysis for the Cord Loop task comparing performances with an instrument before or after completing the task with the other instrument.

**Table 16**: results analysis effect of order of trocar configurations in Mesh Placement task

|  | Sign. |
| --- | --- |
| Time: triangular as first vs second configuration | 0.268 |
| Time: midline as first vs second configuration | 0.427 |
| path length: triangular as first vs second configuration | 0.155 |
| path length: midline as first vs second configuration | 0.301 |
| Maximum force: triangular as first vs second configuration | 0.629 |
| Maximum force: midline as first vs second configuration | 0.460 |
| Average non-zero force: triangular as first vs second configuration | 0.816 |
| Average non-zero force: midline as first vs second configuration | 0.215 |

*Wilcoxon signed-rank

**Table 17**: results analysis effect of order of instruments in Cord Loop task

|  | Sign. |
| --- | --- |
| Time: conventional as first vs second instrument | 0.268 |
| Time: SATA as first vs second instrument | 0.427 |
| path length: conventional as first vs second instrument | 0.155 |
| path length: SATA as first vs second instrument | 0.301 |
| Maximum force: conventional as first vs second instrument | 0.629 |
| Maximum force: SATA as first vs second instrument | 0.460 |
| Average non-zero force: conventional as first vs second instrument | 0.816 |
| Average non-zero force: SATA as first vs second instrument | 0.215 |

*Wilcoxon signed-rank
