# Supplementary material for: A comparative study on trocar configurations and the use of steerable instruments in totally extraperitoneal inguinal hernia surgery training
Source: Surg Endosc. 2025 Feb 3;39(3):2080–90. doi: 10.1007/s00464-025-11541-7 (PMC11870937; doi:10.1007/s00464-025-11541-7)
Supplement: Supplementary file 4 — Supplementary file4 (DOCX 42 KB) [file 464_2025_11541_MOESM4_ESM.docx]

# Supplemental file K: responses to questions regarding comprehensibility and feasibility


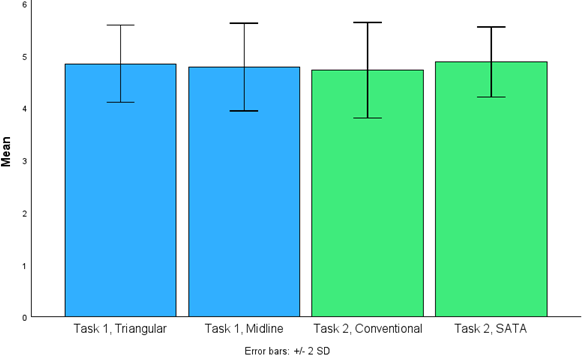


Figure 23: Means of responses to the statement ’ the task was easy to understand’ on a 1-5 Likert scale per task and condition. Mesh Placement task shown in blue, Cord Loop task in green


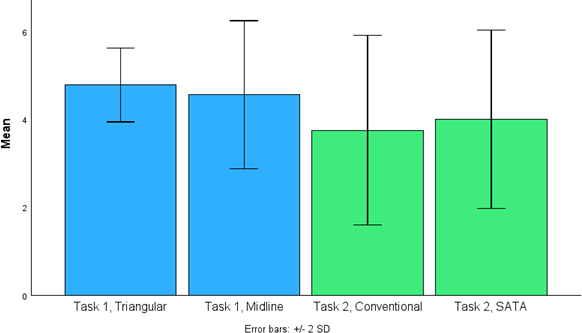


Figure 24: Means of responses to the statement ’ the task was achievable within the given time’ on a 1-5 Likert scale per task and condition. Mesh Placement task shown in blue, Cord Loop task in green
