# Supplementary material for: A comparative study on trocar configurations and the use of steerable instruments in totally extraperitoneal inguinal hernia surgery training
Source: Surg Endosc. 2025 Feb 3;39(3):2080–90. doi: 10.1007/s00464-025-11541-7 (PMC11870937; doi:10.1007/s00464-025-11541-7)
Supplement: Supplementary file 5 — Supplementary file5 (DOCX 14 KB) [file 464_2025_11541_MOESM5_ESM.docx]

# Supplemental file A: registration form

(Translated from Dutch to English)

All collected data will be published anonymously Name: ...........................

Age: ............................

Sex: Male / Female Dominant hand: Left / Right

Study year: Bachelor 1 / Bachelor 2 / Bachelor 3 / Master 1 / Master 2 / Master 3 Study Faculty: Amsterdam / Rotterdam

Experience in laparoscopy (in minutes): ......................

Weekly hours of playing video games (average): ................. Weekly hours of playing a musical instrument (average): ............ In case of playing a musical instrument, which instrument: ...........

I hereby consent to the collection of anonymous data and footage of the training and tests and the use of this data and footage for scientific research.

Date: .............

Signature: .................
