# Supplementary material for: A comparative study on trocar configurations and the use of steerable instruments in totally extraperitoneal inguinal hernia surgery training
Source: Surg Endosc. 2025 Feb 3;39(3):2080–90. doi: 10.1007/s00464-025-11541-7 (PMC11870937; doi:10.1007/s00464-025-11541-7)
Supplement: Supplementary file 6 — Supplementary file6 (DOCX 64 KB) [file 464_2025_11541_MOESM6_ESM.docx]

# Supplemental file B: box trainer panels


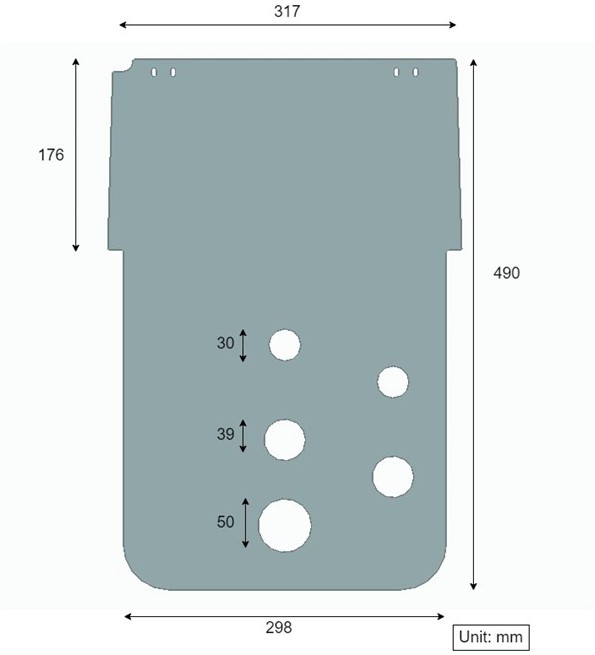

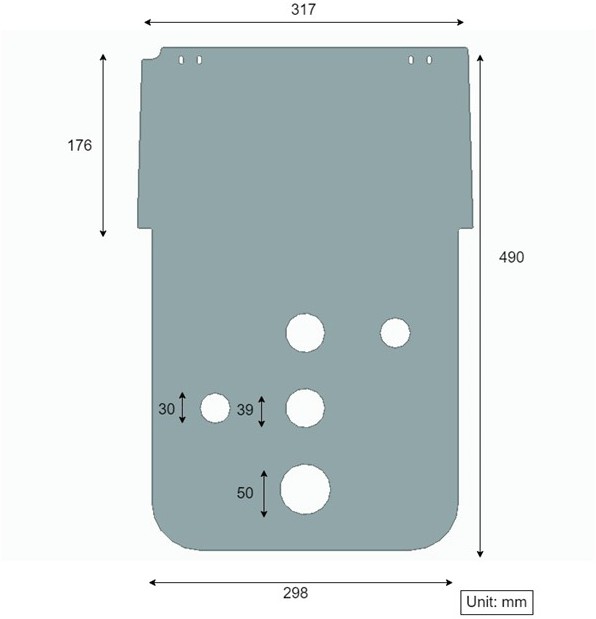


Figure 6: Above: schematic illustration of the panel designed for triangular trocar placement. Below: schematic illustration of the panel designed for midline trocar placement
