# Supplementary material for: A comparative study on trocar configurations and the use of steerable instruments in totally extraperitoneal inguinal hernia surgery training
Source: Surg Endosc. 2025 Feb 3;39(3):2080–90. doi: 10.1007/s00464-025-11541-7 (PMC11870937; doi:10.1007/s00464-025-11541-7)
Supplement: Supplementary file 7 — Supplementary file7 (DOCX 726 KB) [file 464_2025_11541_MOESM7_ESM.docx]

# Supplemental file C: training tasks


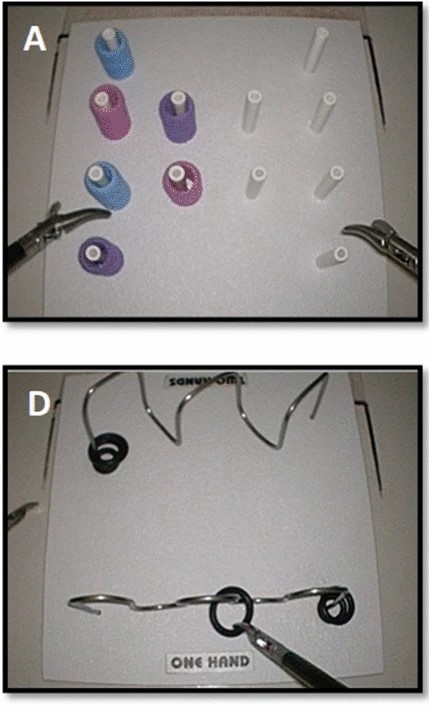


Figure 7: Above: the Post and Sleeve task. Below: the Wire Chaser task.[16](#_bookmark39)

# Mesh Placement task


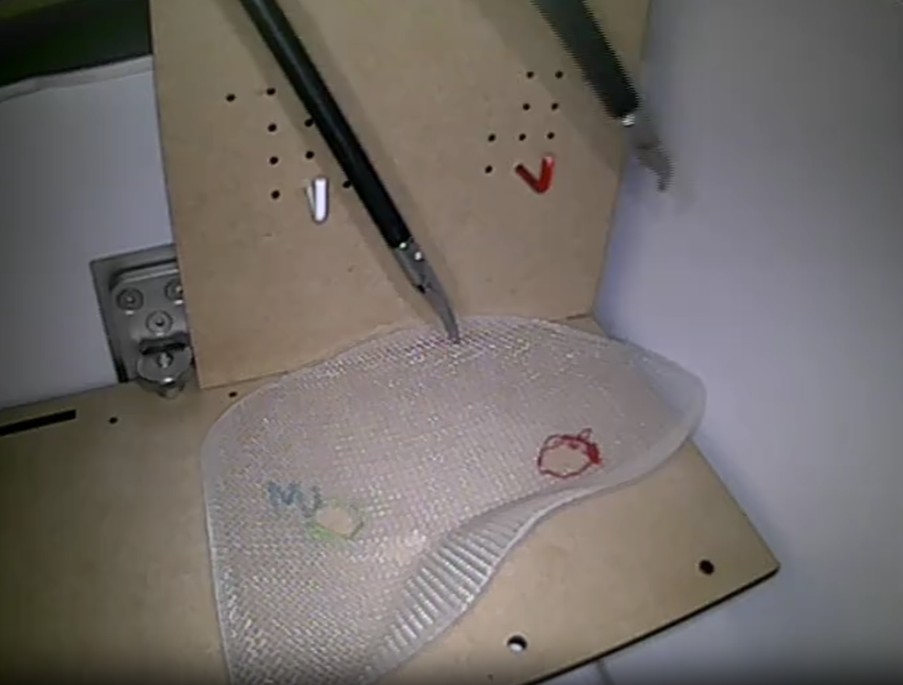


Figure 8: Footage from the camera in the box trainer at the beginning of the Mesh Placement task: with the mesk on the task board


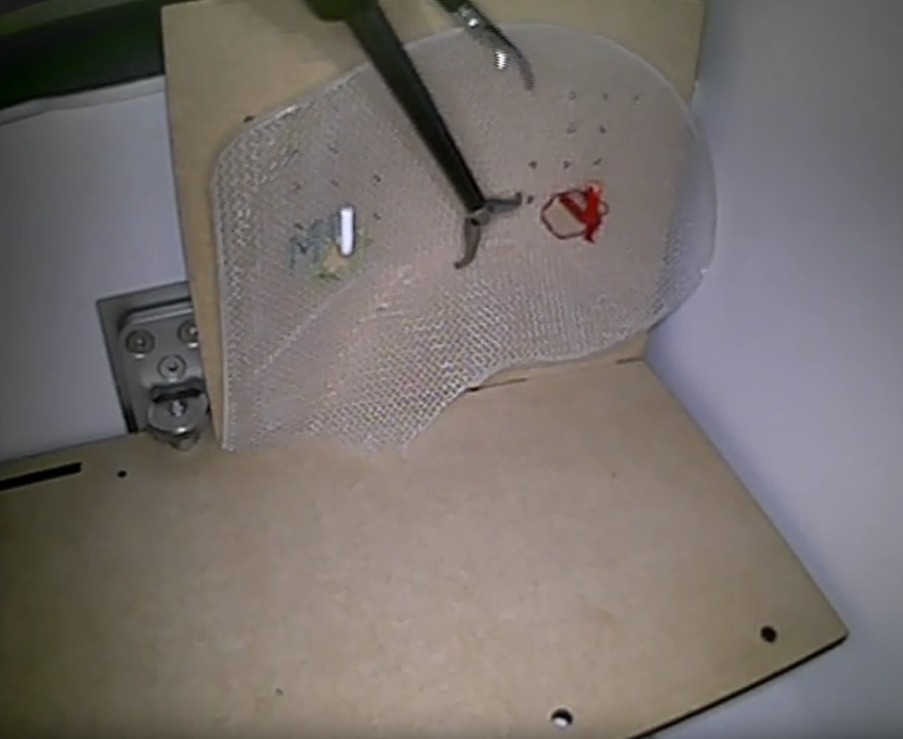


Figure 9: Footage from the camera in the box trainer halfway the Mesh Placement task: with the mesh positioned around the two hooks

# Cord Loop task


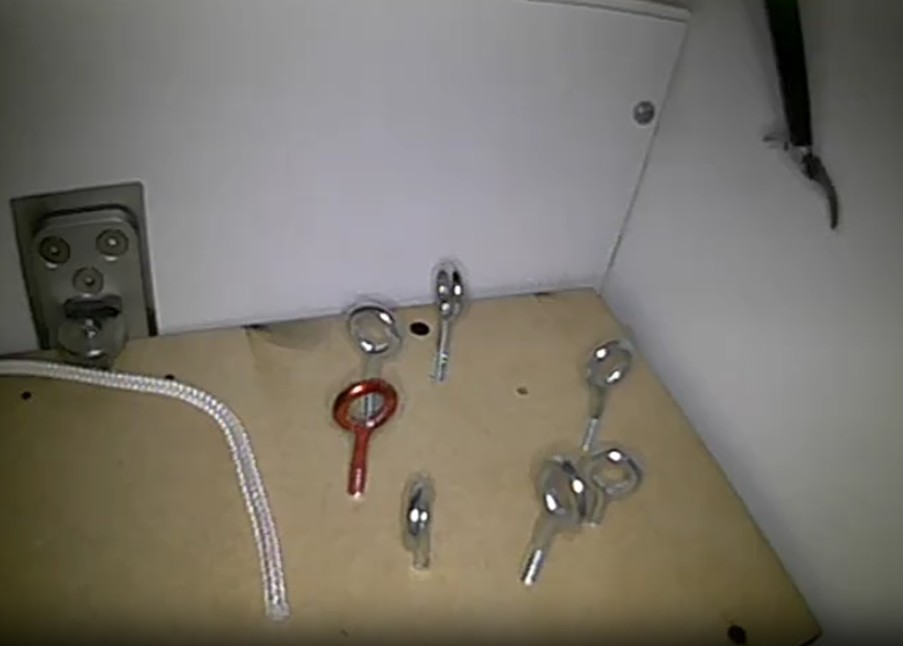


Figure 10: Footage from the camera in the box trainer at the beginning of the Cord loop task: with the cord next to the holes


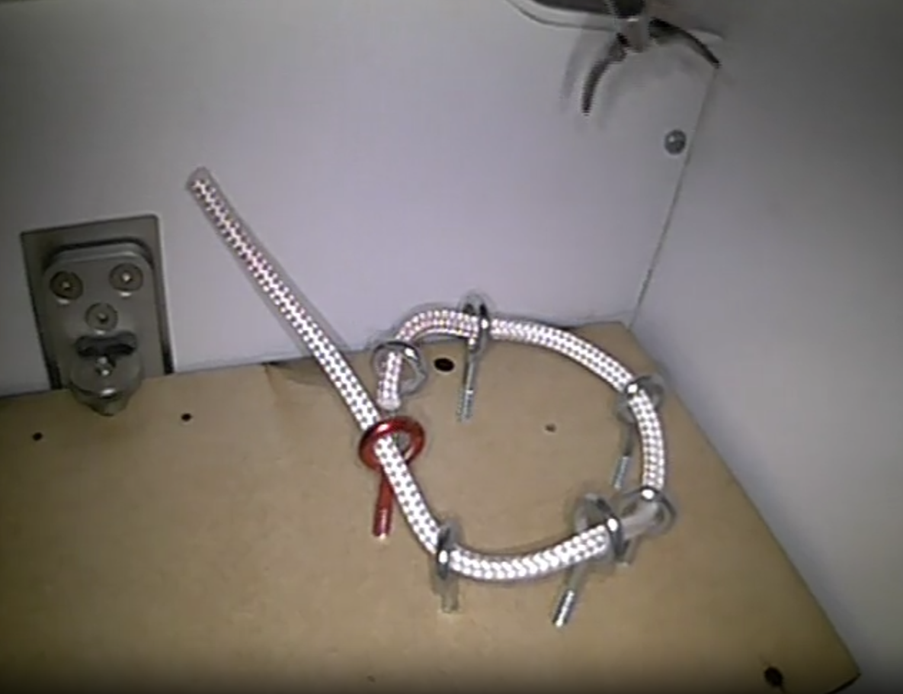


Figure 11: Footage from the camera in the box trainer after completion of the Cord Loop task: with the cord through all seven holes
