# Supplementary material for: A comparative study on trocar configurations and the use of steerable instruments in totally extraperitoneal inguinal hernia surgery training
Source: Surg Endosc. 2025 Feb 3;39(3):2080–90. doi: 10.1007/s00464-025-11541-7 (PMC11870937; doi:10.1007/s00464-025-11541-7)
Supplement: Supplementary file 8 — Supplementary file8 (DOCX 22 KB) [file 464_2025_11541_MOESM8_ESM.docx]

# Supplemental file D: questionnaire for participants

(Translated from Dutch to English)

## Questionnaire after the Mesh Placement task

Please indicate to which extend you agree with the following statements for the task with trocars in triangular configuration

|  | strongly  disagree | disagree | neutral | agree | strongly  agree |
| --- | --- | --- | --- | --- | --- |
| 1. It was easy to perform the task in  this configuration |  |  |  |  |  |
| 2. In this configuration I could easily  keep an overview of my instruments and the task |  |  |  |  |  |
| 3. It is easy to find a comfortable posi-  tion in this setup |  |  |  |  |  |
| 4. The task was easy to understand |  |  |  |  |  |
| 5. Completing the task within the time  limit was achievable |  |  |  |  |  |

Please indicate to which extend you agree with the following statements for the task with trocars in midline configuration

|  | strongly  disagree | disagree | neutral | agree | strongly  agree |
| --- | --- | --- | --- | --- | --- |
| 1. It was easy to perform the task in  this configuration |  |  |  |  |  |
| 2. In this configuration I could easily  keep an overview of my instruments and the task |  |  |  |  |  |
| 3. It is easy to find a comfortable posi-  tion in this setup |  |  |  |  |  |
| 4. The task was easy to understand |  |  |  |  |  |
| 5. Completing the task within the time  limit was achievable |  |  |  |  |  |

## Questionnaire after the Cord Loop task with conventional instruments

Please indicate to which extend you agree with the following statements

|  | strongly  disagree | disagree | neutral | agree | strongly  agree |
| --- | --- | --- | --- | --- | --- |
| 1. It was easy to perform the task with  these instruments |  |  |  |  |  |
| 2. The instruments were easy to use |  |  |  |  |  |
| 3. Using these instruments felt intuitive |  |  |  |  |  |
| 4. The ergonomics of these instruments  feel and work comfortable |  |  |  |  |  |
| 5. The task was easy to understand |  |  |  |  |  |
| 6. Completing the task within the time  limit was achievable |  |  |  |  |  |

## Questionnaire after the Cord Loop task with one SATA and one conventional in- strument

Please indicate to which extend you agree with the following statements

|  | strongly  disagree | disagree | neutral | agree | strongly  agree |
| --- | --- | --- | --- | --- | --- |
| 1. It was easy to perform the task with  these instruments |  |  |  |  |  |
| 2. The instruments were easy to use |  |  |  |  |  |
| 3. With these instruments, I could eas-  ily make precise movements |  |  |  |  |  |
| 4. It is easy to steer the tip of the in-  strument to the desired position |  |  |  |  |  |
| 5. Using these instruments felt intuitive |  |  |  |  |  |
| 6. The ergonomics of these instruments  feel and work comfortable |  |  |  |  |  |
| 7. The use of the steering function was  of added value in this task |  |  |  |  |  |
| 8. The task was easy to understand |  |  |  |  |  |
| 9. Completing the task within the time  limit was achievable |  |  |  |  |  |
