# Supplementary material for: A comparative study on trocar configurations and the use of steerable instruments in totally extraperitoneal inguinal hernia surgery training
Source: Surg Endosc. 2025 Feb 3;39(3):2080–90. doi: 10.1007/s00464-025-11541-7 (PMC11870937; doi:10.1007/s00464-025-11541-7)
Supplement: Supplementary file 9 — Supplementary file9 (DOCX 15 KB) [file 464_2025_11541_MOESM9_ESM.docx]

# Supplemental file E: analysis of baseline characteristics

Table 5 presents the baseline characteristics of the two groups along with the results of the statistical comparison between them.

**Table 5**: Baseline Characteristics of the two study groups and statistical analysis

| Characteristic | Group 1 | Group 2 | Sign. |
| --- | --- | --- | --- |
| Age (years)* | 21 (5) | 21.5 (10) | 0.517 |
| Sex (% female)** | 50% | 75% | 0.273 |
| Dominant hand (% right)** | 93.75% | 81.25% | 0.435 |
| Study year*** | 2 (5) | 2 (5) | 0.783 |
| Laparoscopic experience (min)*** | 0 (60) | 0 (60) | 0.783 |
| Video gaming (hours/ week)*** | 0 (6) | 0 (30) | 0.729 |
| Musical instument (hours / week)*** | 0 (3) | 0 (10) | 0.912 |

Median (range). *unpaired t-test. **Fishers exact test. ***Mann Whitney U.
