# Supplementary material for: A comparative study on trocar configurations and the use of steerable instruments in totally extraperitoneal inguinal hernia surgery training
Source: Surg Endosc. 2025 Feb 3;39(3):2080–90. doi: 10.1007/s00464-025-11541-7 (PMC11870937; doi:10.1007/s00464-025-11541-7)
Supplement: Supplementary file 10 — Supplementary file10 (DOCX 52 KB) [file 464_2025_11541_MOESM10_ESM.docx]

# Supplemental file F: questionnaire responses and analysis

## Trocar placement questionnaire

Tables 6 and 7 show the descriptives and the results of normality tests for the questions on trocar placements. Figure 12 shows the results distribution per question for each configuration.

**Table 6**: Questionnaire responses triangular trocar placement

|  | Mean | Median | Range | IQR | Skewnes | Kurtosis | Norm* |
| --- | --- | --- | --- | --- | --- | --- | --- |
| 1. Easiness task | 4.19 | 4 | 2 | 1 | 0.179 | 0.265 | <0,001 |
| 2. Overview | 4.44 | 4 | 1 | 1 | 0,265 | -2,063 | <0,001 |
| 3. Comfortable posture | 2.94 | 3 | 3 | 1 | 0.518 | -0,126 | <0,001 |
| 4. Understandable task | 4.84 | 5 | 1 | 0 | -1.988 | 2,078 | <0,001 |
| 5. Achievable task | 4,78 | 5 | 1 | 0 | -1,429 | -,039 | <0,001 |

*Normality via Shapiro-Wilk

**Table 7**: Questionnaire responses midline trocar placement

|  | Mean | Median | Range | IQR | Skewnes | Kurtosis | Norm* |
| --- | --- | --- | --- | --- | --- | --- | --- |
| 1. Easiness task | 3.25 | 3 | 2 | 1 | -0.498 | -1.256 | <0.001 |
| 2. Overview | 2.97 | 3 | 3 | 2 | 0.349 | -1.034 | <0.001 |
| 3. Comfortable posture | 4.31 | 4 | 2 | 1 | -0.194 | -0.518 | <0.001 |
| 4. Understandable task | 4.78 | 5 | 1 | 0 | -1.429 | 0.039 | <0.001 |
| 5. Achievable task | 4,56 | 5 | 3 | 1 | -1.777 | 2.044 | <0.001 |

*Normality via Shapiro-Wilk


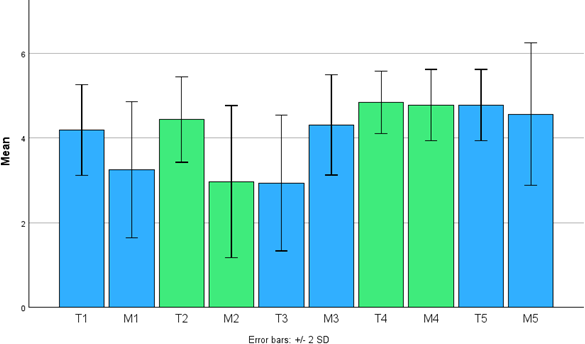


Figure 12: Responses to the questions, shown per question number, for triangular (T) and midline (M) trocar placement

## Instrument questionnaire

Tables 8 and 9 show the descriptives and the results of normality tests for the questions concerning the conven- tional and SATA instruments. Figure 13 shows the results distribution per question for each instrument.

**Table 8**: Questionnaire responses conventional instruments

|  | Mean | Median | Range | IQR | Skewnes | Kurtosis | Norm* |
| --- | --- | --- | --- | --- | --- | --- | --- |
| 1. Easiness task | 3.03 | 3 | 4 | 2 | -0.066 | -0.507 | 0.017 |
| 2. Easiness use instru-  ment | 3.66 | 4 | 3 | 1 | -0.556 | 0.159 | <0.001 |
| 3. Intuitiveness instru-  ment | 3.66 | 4 | 3 | 1 | -0.585 | -0.562 | <0.001 |
| 4. Ergonomics instrument | 3.69 | 4 | 3 | 1 | -0.448 | -0.853 | <0.001 |
| 5. Understandable task | 4.72 | 5 | 1 | 1 | -1.022 | -1.025 | <0.001 |
| 6. Achievable task | 3.75 | 4 | 4 | 2 | -0.619 | -0.124 | 0.002 |

*Normality via Shapiro-Wilk

**Table 9**: Questionnaire responses SATA instruments

|  | Mean | Median | Range | IQR | Skewnes | Kurtosis | Norm* |
| --- | --- | --- | --- | --- | --- | --- | --- |
| 1. Easiness task | 3.50 | 4 | 4 | 1 | -0.722 | -0.334 | <0,001 |
| 2. Easiness use instru-  ment | 3.69 | 4 | 4 | 1 | -1.326 | 1.946 | <0.001 |
| 3. Intuitiveness instru-  ment | 3.56 | 4 | 3 | 1 | -0.203 | -0.513 | 0.001 |
| 4. Ergonomics instrument | 3.75 | 4 | 4 | 1 | -1.075 | 1.703 | <0.001 |
| 5. Understandable task | 4.88 | 5 | 1 | 0 | -2.381 | 3.909 | <0.001 |
| 6. Achievable task | 4 | 4 | 4 | 1 | -1.181 | 1.390 | <0.001 |

*Normality via Shapiro-Wilk


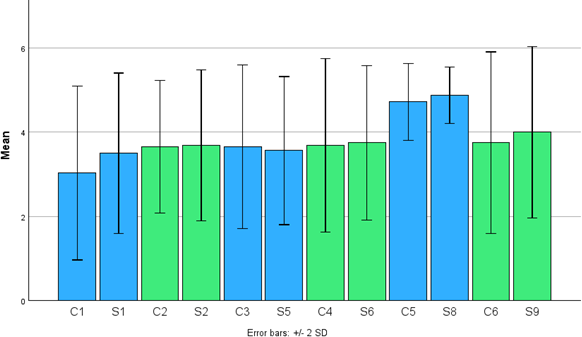


Figure 13: Responses to the questions, shown per question number, for conventional (C) and SATA (S) instru- ments
