# Supplementary material for: A comparative study on trocar configurations and the use of steerable instruments in totally extraperitoneal inguinal hernia surgery training
Source: Surg Endosc. 2025 Feb 3;39(3):2080–90. doi: 10.1007/s00464-025-11541-7 (PMC11870937; doi:10.1007/s00464-025-11541-7)
Supplement: Supplementary file 11 — Supplementary file11 (DOCX 17 KB) [file 464_2025_11541_MOESM11_ESM.docx]

# Supplemental file G: responses to open questions

## Trocar placement in triangular configuration vs. in the midline

**Table 10**: Responses to open questions comparing triangular and midline configuration

| Feedback | Number of mentions |
| --- | --- |
| Triangular: better distinction between left and right instrument | 20 |
| Triangular: easier to orientate | 6 |
| Triangular: instruments cross each other less often | 7 |
| Triangular: easier to estimate depth | 4 |
| Triangular: instruments obstruct the camera view less often | 6 |
| Triangular: feels more intuitive | 1 |
| Midline: a more comfortable posture | 26 |

## Conventional instruments vs SATA instruments

**Table 11**: Responses to open questions comparing conventional and SATA instruments

| Feedback | Number of mentions |
| --- | --- |
| SATA: able to reach more angles | 25 |
| SATA: instruments obstruct the camera view less often | 5 |
| SATA: instruments cross each other less often | 6 |
| SATA: able to manipulate the cord better | 6 |
| SATA: makes the task easier | 5 |
| SATA: end-effector can only rotate 360° which is not enough | 4 |
| SATA: confusion between the two rotary knobs | 3 |
| SATA: ergonomics are uncomfortable | 6 |
| Conventional: ergonomics are uncomfortable | 6 |
| Conventional: manipulating the tip feels more intuitive | 7 |
| Conventional: manipulating the tip requires less thinking | 14 |
| Conventional: more grip on the cord | 1 |
